# Reclassifying stroke lesion anatomy

**DOI:** 10.1016/j.cortex.2021.09.007

**Published:** 2021-12

**Authors:** Anna K. Bonkhoff, Tianbo Xu, Amy Nelson, Robert Gray, Ashwani Jha, Jorge Cardoso, Sebastien Ourselin, Geraint Rees, Hans Rolf Jäger, Parashkev Nachev

**Affiliations:** a J. Philip Kistler Stroke Research Center, Department of Neurology, Massachusetts General Hospital, Harvard Medical School, Boston, USA; b UCL Queen Square Institute of Neurology, University College London, London, UK; c School of Biomedical Engineering & Imaging Sciences, King's College London, London, UK; d Faculty of Life Sciences, University College London, London, UK

**Keywords:** Stroke, Lesion anatomy, Lesion–deficit prediction, Dimensionality reduction, Brain imaging, DWI, diffusion-weighted imaging, t-SNE, t-stochastic neighbour embedding, NMF, non-negative matrix factorization, BA, Brodmann Area

## Abstract

Cognitive and behavioural outcomes in stroke reflect the interaction between two complex anatomically-distributed patterns: the functional organization of the brain and the structural distribution of ischaemic injury. Conventional outcome models—for individual prediction or population-level inference—commonly ignore this complexity, discarding anatomical variation beyond simple characteristics such as lesion volume. This sets a hard limit on the maximum fidelity such models can achieve. High-dimensional methods can overcome this problem, but only at prohibitively large data scales. Drawing on one of the largest published collections of anatomically-registered imaging of acute stroke—N = 1333—here we use non-linear dimensionality reduction to derive a succinct latent representation of the anatomical patterns of ischaemic injury, agglomerated into 21 distinct intuitive categories. We compare the maximal predictive performance it enables against both simpler low-dimensional and more complex high-dimensional representations, employing multiple empirically-informed ground truth models of distributed structure–outcome relationships. We show our representation sets a substantially higher ceiling on predictive fidelity than conventional low-dimensional approaches, but lower than that achievable within a high-dimensional framework. Where descriptive simplicity is a necessity, such as within clinical care or research trials of modest size, the representation we propose arguably offers a favourable compromise of compactness and fidelity.

## 1 Introduction

**Fig. 1:**
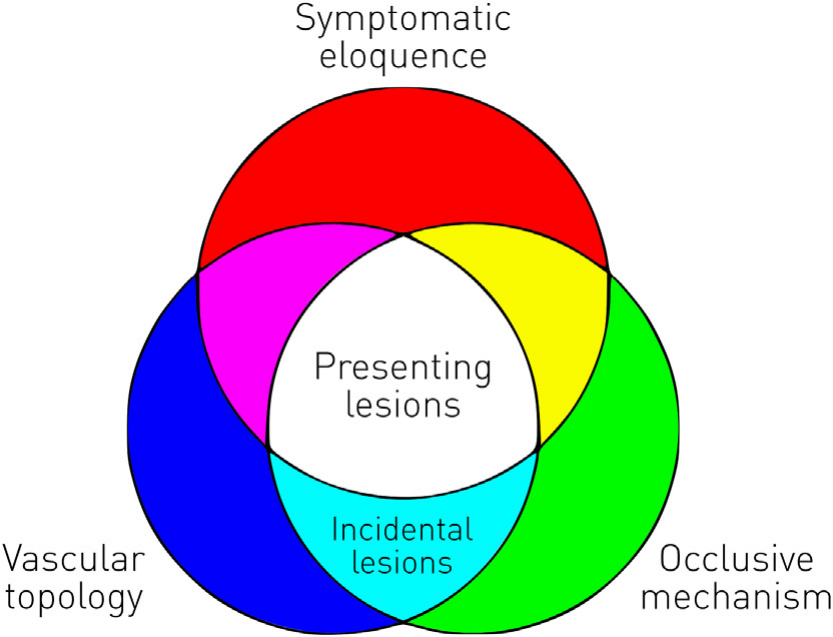
**The causal triad of stroke lesion anatomy.** The spatial features of acutely presenting stroke lesions are generally determined by the interaction of three factors: the vascular topology (blue), the occlusive mechanism (green), and the symptomatic eloquence of the damaged brain (red). Incidental lesions (cyan) are free of the last constraint. Niche cases are global hypoperfusion (magenta) that need not involve focal occlusion or stenosis but will be shaped by vascular topology, and cardiogenic embolic “showers” (yellow) too small to be materially influenced by the structure of the vascular tree.

Stroke is remarkable in the wide diversity of its cognitive and behavioural manifestations and the difficulty of predicting them from the contemporaneous clinical picture alone (Boyd et al., 2017; Stinear, 2017; Ward, 2017). This cardinal aspect impedes the management of individual patients, the identification of protective or exacerbating factors in the population, and the quantification of treatment doses and effects. Were this heterogeneity biologically impossible to capture, we could do no more than to accept it as an unalterable fact of life. But it arises from the interaction of two biological characteristics that are, at least in theory, accessible even if complex enough to *appear* suffused with randomness. The first is the *functional anatomy of the brain* focal ischaemic injury definitionally disrupts, now comprehensively established to be not only highly complex but also remarkably consistent across individuals: meta-analytic imaging databases would otherwise be filled with noise, not generalisable clusters of coherent activation (Biswal et al., 2010; Eickhoff, Constable, & Yeo, 2018; Glasser et al., 2016). The second is the *structural anatomy of stroke*: the product of pathological and anatomical factors that are plausibly *both* highly complex *and* non-random (Adams Jr et al., 1993; Amarenco, Bogousslavsky, Caplan, Donnan, & Hennerici, 2009; Mah, Husain, Rees, & Nachev, 2014). The topology of the vascular tree, the mechanisms of occlusion or rupture, and the symptomatic eloquence^2^ of damaged brain will all combine to generate elaborate *patterns* of focal injury that will nonetheless conform to a potentially knowable spatial distribution (Fig. 1). Since our knowledge of the functional anatomy of the brain depends to a great extent on the study of the functional consequences of stroke (Adolphs, 2016; Damasio & Damasio, 1989; Rorden & Karnath, 2004), the second of these characteristics is arguably of prior importance, and is our specific concern here.

Now the first question when confronted with any complex biological pattern is this: how do we identify a succinct *representation* that simplifies the pattern while preserving detail critical to modelling the biological system in which it occurs? Simplicity is desirable for two inter-related reasons: first, to render the characterisation of each instance perspicuous—easily apprehensible—so that its application may be readily intelligible (intuitively understood), practicable (easily implemented), and reproducible (replicable across time and context). Second, to enable robust, objective comparisons between related instances—especially in observational and interventional studies—where a paucity of variables improves statistical power and generalisability by reducing the risk of overfitting (James, Witten, Hastie, & Tibshirani, 2013). Preservation of detail is desirable for two roughly corresponding reasons: first, to maximise predictive and inferential accuracy in individuals—of value in tailoring clinical practice to a patient's specific needs—and second, to permit a closer model fit to the population—of value in research studies by explaining more of the observed variability (Shmueli, 2010).

Since simplicity and detail inevitably stand in opposition, our task is to identify a compromise between the two. How should such compromise be weighted? From a clinical perspective, individual-level accuracy should be more important than perspicuity: clinical outcomes matter more than our intellectual satisfaction with the means of achieving them (Holm, 2019; Rajkomar, Dean, & Kohane, 2019). From a research perspective, achieving a closer model fit—provided it generalises to unseen data—should be more important than maximizing statistical power to estimate model parameters, for our confidence that the model we are testing is closer to the generative process is thereby enhanced. Though we should seek to optimise both, predictive fidelity ought to take precedence over simplicity in selecting candidate succinct representations (Yarkoni & Westfall, 2017).

Such an optimum is best derived *directly* from large-scale lesion data, without strong prior beliefs about its constitution, to reduce bias and to bring non-intuitive solutions into play. It is also best derived from lesion data *alone* in the first instance, even if its utility is to be ultimately established by its power to predict outcomes in downstream discriminative models (Corbetta et al., 2015; Ramsey et al., 2017). This is because a representation explicitly steered by a specific discriminative objective—predicting motor disability, for example—may be distorted by it, impairing generalisability when deployed in the context of other predictive tasks. Equally, the *first* test of the potential clinical utility of a representation is *not* its performance on real patient outcomes but its performance on the combination of real lesion data with synthetic lesion-deficit models. This is because the fidelity of any prediction can only be objectively quantified against a hard functional–anatomical ground truth where the relation between focal damage and outcome is definitively known by being explicitly prescribed. With actual patient outcomes, since the true lesion-deficit relation is unknown, any general comparison between representations will be obscured both by non-anatomical factors such as the global state of the brain and anatomical factors peculiar to the specific outcome at hand.

A cardinal characteristic of any representation is its dimensionality: the number of variables used to describe each instance. The source dimensionality of stroke lesions is equal to the number of independently sampled locations within the brain: typically many thousands with modern imaging. Since it is difficult to grasp a representation of higher dimensionality than two, to satisfy the requirement of perspicuity we must attempt to derive one- and two-dimensional representations as our primary focus, evaluating moderately higher dimensionality for comparison.

Our study therefore adopts the following approach. We apply unsupervised learning to one of the largest published collections of registered lesion maps of acute ischaemic stroke imaged with diffusion weighted magnetic resonance imaging (Xu, Rolf Jäger, Husain, Rees, & Nachev, 2017), yielding categorial, 2-dimensional, and 50-dimensional representations that optimize the preservation of high-dimensional similarities and differences in the patterns of injury. Defining a comprehensive set of hypothetical lesion-deficit relations based on functionally-informed structural parcellations of the whole brain, we then explicitly compare the predictive fidelity of these representations against a simple volume-based parameterisation of each lesion. Two sets of optimized low-dimensional representations—categorial and 2-dimensional—are thus compared against two baselines: a conventional, simple low-dimensional, and an illustrative high-dimensional representation. The potential benefit of seeking jointly optimised representations of stroke patterns is thereby quantified.

## 2 Materials & methods

### 2.1 Patients

We identified a set of 1333 patients admitted between 2001 and 2014 to University College London Hospitals (UCLH) with a clinical diagnosis of acute ischaemic stroke confirmed by diffusion weighted imaging (DWI). Since DWI was routinely performed on the majority of attending patients, the sample was representative of the population, constrained mostly by contraindications to and tolerability of MRI. Age ranged from 18 to 97 years, mean 63.89, standard deviation 15.91; the proportion of males was .561; ethnicity was representative of London (Supplementary Figures 1 and 2). The inclusion criteria were age 18 and above, a clinical diagnosis of acute ischaemic stroke, and the presence of a segmentable acute ischaemic lesion on diffusion weighted imaging conducted within 10 days of clinical presentation. The exclusion criteria were the presence of additional non-ischaemic pathology that substantively distorted the anatomy of the ischaemic lesion and/or rendered its anatomical registration inaccurate on neuroradiological inspection. Both inclusion and exclusion criteria were set prior to analysis. All manipulations, and all measures in the study, are stated below. The study was performed under ethical approval by the local research ethics committee for consentless use of fully-anonymized data. The majority of the data has been previously published in another study (Xu et al., 2017). No part of the study procedures was pre-registered prior to the research being conducted.

### 2.2 Imaging

#### 2.2.1 Data acquisition

All acquisitions were performed on scanners manufactured by General Electric (Genesis Signa), Philips (Achieva and Ingenia), or Siemens (Avanto, Skyra and Verio) with field strength of either 1.5 or 3 T. This diversity reflects changes in routine clinical practice over the period of data collection rather than differences in individual indications. All scans were obtained as part of the clinical routine, employing clinical protocols. We extracted from each imaging study the echoplanar DWI for lesion segmentation and inter-subject registration. DWI is widely used to detect and locate acute ischemic lesions (Fiebach et al., 2002). In its clinical application, it consists of an image with a b value of 0 sec/mm^2^ that is relatively insensitive to acute ischaemia but shows reasonable tissue contrast, and an image with a b value of 1000 sec/mm^2^ that is sharply sensitive to ischaemia but has poor normal tissue contrast. This complementarity can be exploited to achieve both good lesion segmentation—which depends on the contrast between lesioned and normal tissue—and good brain registration—which depends on the contrast between normal tissue types. Note that the spatial scale and contrast-to-noise ratio of diffusion weighted imaging *in the context of lesion modelling* is such that instrument variability has plausibly little impact on the analysis. This is reflected in the widespread use of instrumentally heterogeneous imaging in lesion studies.

#### 2.2.2 Image processing

A fully-automated algorithm, described in detail in (Xu et al., 2017) and reproduced in Supplementary Material, generated a binary lesion mask in Montreal Neurological Institute (MNI) stereotactic space, sampled at 2 mm isotropic resolution. In brief, a custom set of MATLAB routines based on SPM12 (http://www.fil.ion.ucl.ac.uk/spm/software/spm12/) were used to co-register each b0 and b1000 pair, derive from the b0 a non-linear deformation field to MNI space and apply it to the b1000 (Ashburner & Friston, 2005), and segment the lesion using the anomaly metric, *zeta* (Mah, Jager, Kennard, Husain, & Nachev, 2014; Xu et al., 2017), yielding a whole-brain, voxel-wise, binary map of ischaemic damage, resliced at 2 mm^3^ resolution. Supplementary Figure 3 shows the average of all lesions.

### 2.3 Deriving a succinct lesion representation

To maximise coverage and account for natural spatial variation, the stack of registered binary lesion images was collapsed onto one hemisphere and smoothed by a Gaussian filter of 2 mm full width at half maximum. Though there are isolated, idiosyncratic structural asymmetries, the macroscopic vascular organisation of the brain is symmetric, with no empirical evidence—published or inherent in clinical practice—for any *systematic* lateralisation across the population. Given our focus on the *cardinal* aspects of the lesion distribution, plausibly driven by anatomical characteristics that are evidently non-lateralised, it is reasonable to model under the assumption of symmetry.

Non-negative matrix factorisation (Lee & Seung, 1999), was then used to embed the 902 629 dimensions of the images into a 50 dimensional space, yielding our 50-dimensional representation. This approach is preferable to principal component analysis here for two reasons: first, because the input elements are exclusively positive, and second, because a parts-based decomposition is more likely to achieve good separation between lesion patterns. The value of 50 was chosen as an intuitively non-apprehensible dimensionality substantially higher than the two-dimensional and lower representations for which we need a high dimensional contrast.

To derive a two-dimensional representation we applied *t*-distributed stochastic neighbour embedding to the 50-dimensional representation (as commonly practised to ensure stability) rather than the raw data, with a perplexity setting of 30 (Maaten & Hinton, 2008)). The rationale for using t-SNE—a non-linear dimensionality reduction method with established state-of-the-art performance on many biological datasets (Abdelmoula et al., 2016; Amir et al., 2013; Shekhar, Brodin, Davis, & Chakraborty, 2014)—is that conventional linear methods cannot capture the hierarchical patterns of dependence the fundamental nature of the brain's blood supply imposes on the data. T-SNE has the further advantage of preserving similarities and differences at multiple spatial scales, another inevitable feature of patterns predominantly shaped by the vasculature. The resultant two-dimensional representation was further refined by structure-aware filtering ((Wu et al., 2018), regularization power mu = .2, and neighbourhood size *r* = .1).

Finally, the resultant two-dimensional representation was discretized by Ward hierarchical agglomerative clustering into a categorial representation. The choice of clustering algorithm was motivated by the natural hierarchical structure of the vascular tree, and by the absence of any requirement to specify the desired number of clusters (J. Friedman, Hastie, & Tibshirani, 2001; Ward, 1963). We chose a threshold of 30 clusters as a reasonable compromise between compactness and spatial granularity. Each cluster was subsequently evaluated for redundancy by an experienced neuroradiologist (HRJ) and a neurologist (PN), pruning the final clustering to 21 distinct clusters by amalgamation. Voxel-wise averages across the members of each cluster yields a set of “centroid” archetypal images that capture the distinctive characteristics of the category in anatomical space.

#### 2.3.1 Alternative succinct lesion representations

To investigate the potential capability of other techniques for reducing dimensionality to the initial, two-dimensional succinct representation, we replicated the preceding processing pipeline with the substitution of Principal Component Analysis (PCA) alone, PCA followed by t-SNE, Uniform Manifold Approximation and Projection (UMAP) (McInnes et al., 2018) alone, PCA followed by UMAP, and NMF followed by UMAP. We focused on UMAP as a new general non-linear dimension reduction technique, closely related to t-SNE, that has the advantage of being deterministic, but has yet to see widespread use. The alternative representations were qualitatively compared with the categorial labels from our one-dimensional representation.

#### 2.3.2 Ground truth lesion–deficit maps

Our objective is to quantify the potential utility of our lesion representation in predicting patient functional outcomes. Predictive performance here depends on two interacting factors: the underlying lesion–deficit relation, and the capacity of the lesion representation to make use of it within discriminative models used to forecast future outcomes. Since our interest here is in the second of these two factors, *we must fix the first by positing a hypothetical lesion*–*deficit map.* Otherwise performance estimates will be unquantifiably and unpredictably distorted by error and uncertainty in the underlying real-world relation between lesions and their associated deficits.

The posited hypothetical lesion–deficit mapping must nonetheless be biologically plausible. It must also afford coverage of the entire brain, for generalisation from one anatomical region to another cannot be assumed. Finally, more than one map is desirable to reassure us the result is not an accidental artefact of the specific choice of ground truth.

We therefore created two sets of ground truth lesion–deficit maps. The first set was defined by damage to at least 15% of a sub-network of functionally related Brodmann areas (BA) and their underlying white matter as specified in Chris Rorden's widely-used template distributed with MRIcro (http://www.mccauslandcenter.sc.edu/mricro/index.html). These maps included Brodmann areas implicated in visuospatial neglect (BA 39/44), picture naming (BA 37/38), sensorimotor areas (BA 6, 4a/b, 3 a/b, 1,2; adopted by (Rehme et al., 2015)), visual (BA 17, 18, 19), and speech areas (BA 22, 39, 40, 44, 45). These functional systems were chosen as amongst the most frequently affected following stroke (Kelly-Hayes et al., 2003) (Gall et al., 2010), and as modelled in previous lesion-deficit simulation studies (visuospatial neglect, picture naming (Mah et al., 2014) (Sperber, 2020)), facilitating comparison. Furthermore, the underlying patterns of neural dependence span multiple vascular territories, enabling more comprehensive testing of the predictive capacities of our lesion embeddings and strengthening generalisation across diverse predictive tasks and contexts. A subcortical component was additionally introduced with Archer's sensorimotor tract template (Archer, Vaillancourt, & Coombes, 2018), analogously defining a deficit where the lesion includes at least 15% of a given tract. A sub-network was treated as “affected” if at least one of its constituent areas was lesioned above the critical threshold (Supplementary Figure 5).

The second set of maps exploited Schaefer's recent whole brain parcellation based on resting state functional MRI data (Schaefer et al., 2017), combined with Yeo's 17-network parcellation (Buckner, Krienen, Castellanos, Diaz, & Yeo, 2011). Here each of Schaefer's 100 regions was assigned to its corresponding Yeo functional sub-network. As before, we defined a sub-network as “affected” if at least 15% of the voxels of at least one region within it were lesioned (Supplementary Figure 6). An exhaustive list is given in Supplementary Table 1.

#### 2.3.3 Predicting deficits

The foregoing lesion–deficit maps enabled us to define a lesion as being associated with a deficit or not, for each of the given functionally informed anatomical networks, yielding a ground truth against which predictions with models employing different lesion representations could then be tested. Four different representations were evaluated. The simplest, “Baseline” representation was constituted of the age of the patient and the volume of the lesion. The next two representations were based on age and lesion volume as well as our low dimensional embeddings: the Ward cluster membership of the lesion (“Categorial”), and the two-dimensional t-SNE coordinates (”2D”). The final representation was the 50-dimensional NMF decomposition (”50D”). Note the purpose of the 50-dimensional representation is to provide a high-dimensional contrast far removed from plausible intelligibility rather than to establish the maximum achievable within a model unconstrained in its input dimensionality. Such a maximum would depend not only on the representation but also on the optimality of the predictive modelling architecture and its tuning, and its supportability by the available lesion data, making it hard to draw any conclusions about the marginal contribution of the representation itself: the focus of our study. We therefore did not explore higher-dimensional models, including those operating at voxel level.

Though low-dimensional, these representations do not linearise the relation to hypothetical deficits. We therefore chose a flexible, non-linear architecture for predictive modelling: gradient boosting machines (GBM) (J. H. Friedman, 2001). For each set of network ground truths and lesion representations, randomly resampling the dataset with equally balanced draws from the “affected” and “unaffected” contingents, we iteratively trained and tested on separate subsets ten-fold, yielding estimates of the mean performance and its variability. The hyper-parameters of the trained models—loss (“deviance” vs “exponential”), number of estimators (100, 300, or 500), and maximal tree depth (1, 2 or 3)—were optimised through five-fold nested cross-validation. The primary measure of performance was accuracy. Note the balanced sampling means this is also the balanced accuracy: chance is set at 50%. Ancillary analyses using instead the area under the receiver operating curve (AUROC) are given in Supplementary Material, as is an outline of the entire workflow (Supplementary Figure 4).

### 2.4 Statistical analysis

The recommended approach to quantifying the robustness of differences in predictive performance achievable with the four different representations is by cross-validation 95% confidence intervals on the balanced accuracy and AUROC measures: this is standard in the evaluation of complex multivariate models (Varoquaux et al., 2017). For those nonetheless accustomed to more conventional tests, we added two-way ANOVAs, separately for each of the two sets of lesion-deficit maps. We examined the main effects of the lesion representation type (“Baseline”, “Categorial”, “2D” or “50D”) and the sub-network (different for each of the two sets of lesion–deficit maps), as well as their interaction. The F-statistic was quoted and the level of significance was set at .05. No part of the study analysis was pre-registered prior to the research being conducted.

### 2.5 Data and code availability

The lesion maps employed in this study are available from the corresponding authors on request by email. Analyses (NMF (Lee & Seung, 1999) (Cichocki & Phan, 2009) (Févotte & Idier, 2011), t-SNE (Maaten & Hinton, 2008), GBM (J. H. Friedman, 2001)) were primarily performed in a Python 3.7 Jupyter Notebook framework (relying on SciKit-learn .19 (Pedregosa et al., 2011)). Example code for the automated derivation of a low-dimensional stroke lesion representation can be found here: https://github.com/AnnaBonkhoff/Reclassifying_stroke_lesion_anatomy. Lesion maps of the final 21 archetypal clusters in MNI-space can be downloaded here: https://github.com/AnnaBonkhoff/Reclassifying_stroke_lesion_anatomy. Any new, test lesion can be assigned to its closest archetype by quantifying its comparative similarity on a suitable binary distance metric. The code for structure-aware filtering is openly available here: https://codeocean.com/capsule/1845868/tree/v1.

## 3 Results

### 3.1 A succinct representation of ischaemic stroke

**Fig. 2:**
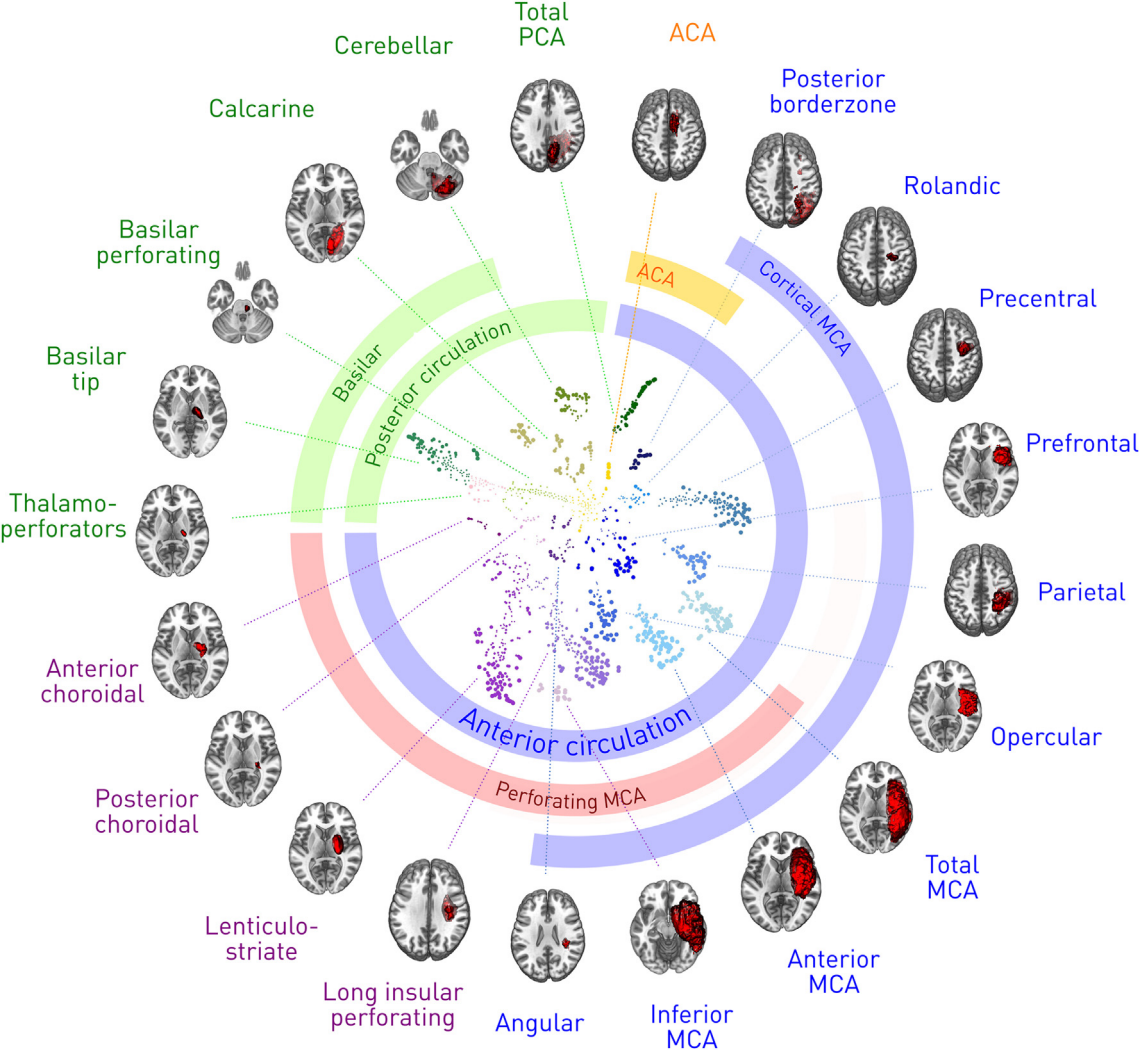
**Two-dimensional representation and clustering of focal ischaemic lesions.** Displayed as a scatter-plot in Cartesian latent dimensions (axes not shown) are the two-dimensional representations of each of the 1333 lesions, with point size proportional to lesion volume. Lesions with similar anatomical features are rendered proximal in this latent space in proportion to their similarity, yielding a set of natural clusters formalised with Ward hierarchical clustering into 21 distinct categories (coloured the same) plausibly related to the underlying vascular tree (coloured rings). Volumetric representations of the average lesion of each cluster—effectively the centroid—are shown in the periphery, centred on the most informative slice. Each category of cluster is given an identifying name for classification purposes. Note all data is collapsed onto one hemisphere for simplicity.

**Fig. 3:**
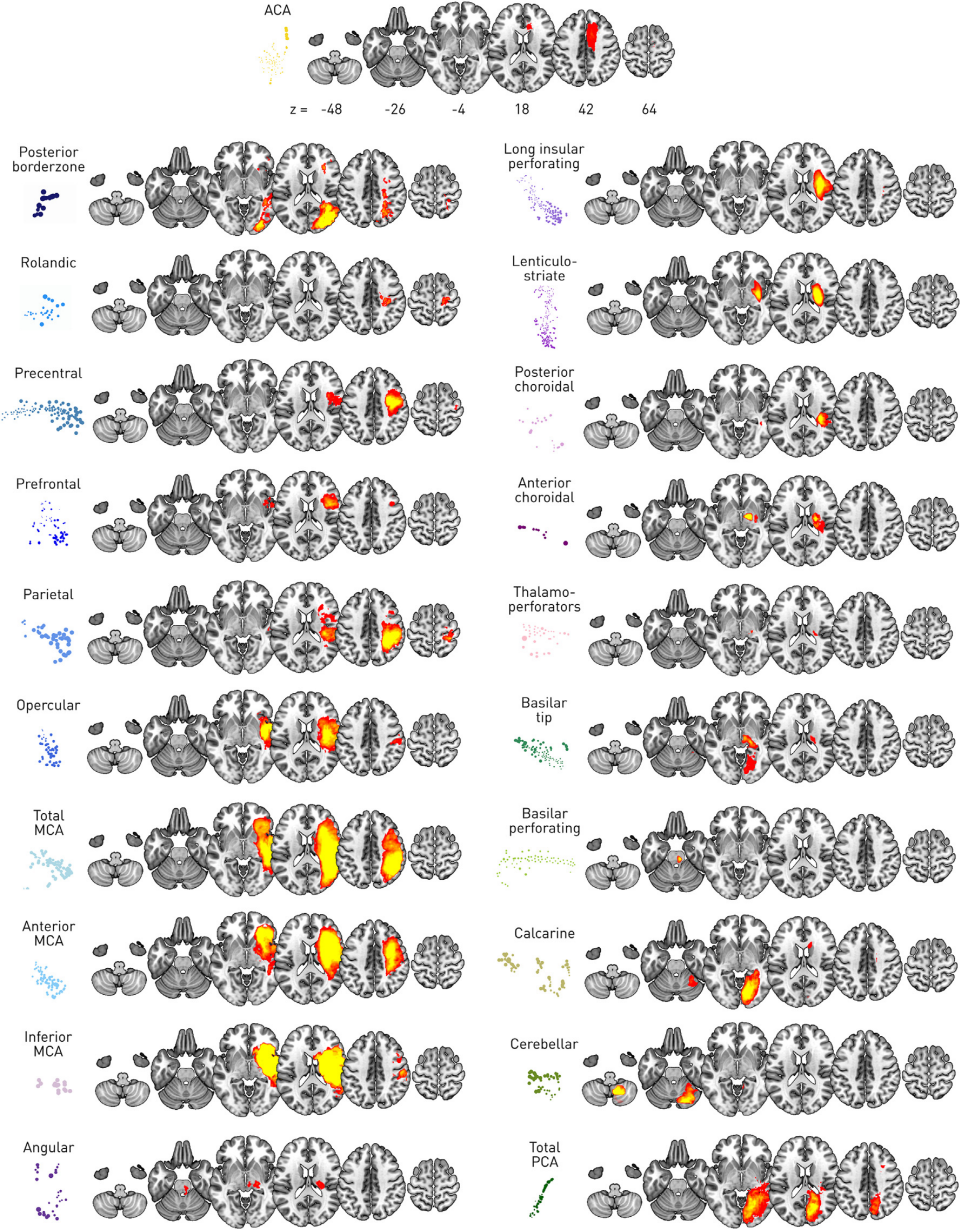
**Detailed anatomy of the categorial lesion representation.** The archetypal centroid of each cluster from the two-dimensional embedding (displayed on the left of each column row) is displayed overlaid on an illustrative normal brain image in Montreal Neurological Institute stereotactic space at the *z* axis locations given in the first row.

The succinct lesion representation shows a clear subdivision into 21 stereotyped clusters (Fig. 2, centre). The archetypal centroids of these clusters, projected into anatomical space, conform to patterns that are plausibly the joint outcome of vascular topology, occlusive mechanisms, and symptomatic eloquence (Fig. 2, periphery). Detailed maps of the centroids are displayed in Fig. 3.

### 3.2 Predictive performance

**Fig. 4:**
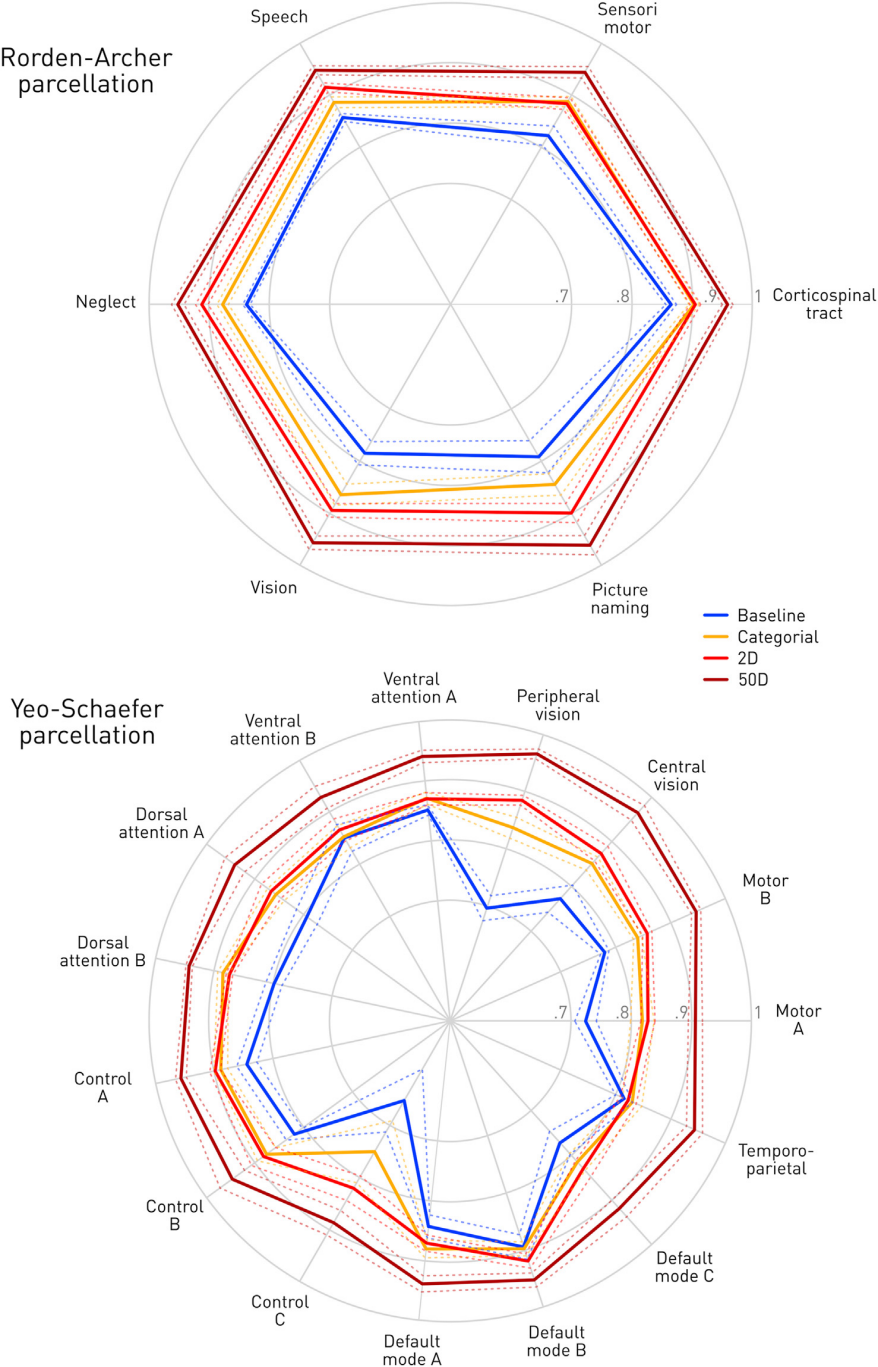
**Quantification of simulated behavioural outcome predictive performance.** For each of four incrementally enriched representations—baseline age and lesion volume (blue), cluster membership (orange), two-dimensional representation coordinates (red), and 50-dimensional NMF representations coordinates (claret)—achieved balanced accuracy is depicted as a spider-plot across individual areas within the Rorden-Archer parcellation (top), and the Yeo-Schaefer parcellation (bottom). Dotted lines identify 95% confidence intervals from the cross-validation procedure. The origin of the spider indicates prediction at chance level (50%); outer circles indicate 70%, 80% and 90% accuracy. Note that predictive accuracy generally increases with dimensionality but that the categorial representation performs substantially better than age and lesion volume alone.

Compared with the baseline, the succinct representation achieved substantially greater than baseline cross-validated predictive accuracy across both sets of lesion-deficit maps and all sub-networks (Fig. 4, Supplementary Tables 1 and 2). The Rorden-Archer models relying only on patient age and lesion size (“Baseline”) achieved a mean cross-validated prediction accuracy of .83(±.01 95%CI), whereas models additionally based on cluster membership (“Categorial”) or two-dimensional embedded coordinates (”2D”) achieved accuracies of .88(±.01 95%CI) and .90(±.01 95%CI), respectively. The corresponding numbers for Yeo-Schaefer models were accuracy of .79(±.01 95%CI) for “Baseline”, .85(±.01 95%CI) for “Categorial”, and .86(±.01 95%CI) for “2D”.

The observed performance also varied with the target functional anatomy. Across the Rorden-Archer models, vision and picture naming yielded larger benefits (10.8–10.9%) than the rest (4.0–7.4%). The highest scores were generally obtained for speech deficits and neglect with an accuracy of .92(±.01 95%CI) and .91(±.02 95%CI), respectively, for the model incorporating information from t-SNE coordinates (”2D”). Across the Yeo-Schaefer models, motor A, peripheral & central vision, and Control C network were most rewarding (10.1–18.9%), with a maximal improvement in case of peripheral vision. The best accuracies were found for the Default mode B network with .92(±.02 95%CI) and Control A network .90(±.02 95%CI) for “2D” model.

Neither succinct representation, however, matched the 50-dimensional representation's test accuracy of .95(±.01 95%CI) for Rorden-Archer and .98(±.002 95%CI) for the Yeo-Schaefer models. This also showed less predictive variability across models and parcellations.

Analysis of AUROC measures yielded an essentially identical picture (Supplementary Figure 7; Supplementary Tables 1 and 2).

ANOVAs performed separately for the Rorden-Archer and Yeo Schaefer parcellations revealed significant main effects of “Representation” (Two-way ANOVA: F-Statistic = 352.2, *p* << .001, Rorden-Archer; F-Statistic = 558.0, *p* << .001, Yeo-Schaefer) and “Deficit” (F-Statistic = 17.0, *p* << .001, Rorden-Archer; F-Statistic = 46.5, *p* << .001, Yeo-Schaefer). There was an interaction between the two factors: F-Statistic = 6.5, *p* << .001, Rorden-Archer; F-Statistic = 8.1, *p* << .001, Yeo-Schaefer, c.f. Supplementary Tables 3 and 4).

### 3.3 Alternative representations

Though our objective is not to compare different possible succinct representations but to quantify the potential predictive superiority of a well-crafted succinct representation against the simple baseline models in current use, we provide a qualitative illustration of the separation between clusters achievable with other techniques, with our categorial labels provided as a reference (Supplementary Figure 8).

## 4 Discussion

We have derived a representation of ischaemic lesions—drawn from one of the largest published collection of registered stroke images—that is almost as succinct as conventional lesion metrics while enabling substantively greater predictive power. Our purely data-driven approach integrates the influence of topological, mechanistic, and symptomatic drivers of the stereotypy of acutely presenting lesions, yielding a comprehensive, generalisable reclassification of the anatomy of ischaemic stroke. Membership of 21 archetypal clusters, and the coordinates within a two-dimensional embedded latent space, distil anatomical information in readily interpretable form. Each cluster is readily explicable within the causal triad of stroke lesion patterns—vascular topology, occlusive mechanism, and symptomatic eloquence—facilitating the intuitive assignment of a lesion to its category. Both the two-dimensional and categorial representations are succinct enough to be handled by relatively simple predictive models powered by modest quantities of data, for any predictive task, making their use in downstream modelling readily practicable. The clear disentanglement of spatially distinct categories assures reproducibility, for membership is determined by strongly differentiated anatomical characteristics plausibly stable across instrumental and wider brain structural variations.

The principal value of this representation is as a simple “drop-in” replacement for the anatomical classifications of stroke in current use, across both observational and interventional studies. Since it is derived *independently of any predictive task,* it is not biased in favour or against any specific clinical context, assuring strong generalisability. An anatomical classification derived from a predictive model—long-term motor recovery, for example—would be inevitably biased by the critical anatomical decision boundary in the brain, limiting its wider utility. Indeed, explicit guidance by an outcome decision boundary always magnifies the risk of overfitting, a problem the field of representation learning (Bengio, Courville, & Vincent, 2013) has in part emerged to solve. The use of lesion properties *alone* to derive the representation, *without any supervision*, from a large, essentially unselected dataset, ensures equal applicability to any predictive task. We make the 21 archetypal lesion categories available to facilitate use in downstream research and clinical settings.

Equally, the ground truth models quantifying the relative predictive power must be synthetic here, even if guided by empirically-derived parcellations of the brain. Real patient outcomes for a dataset of this size would either be too coarse—such as mRS—or limited to a narrow range of functional domains, limiting generalisability. Moreover, no objective quantification of the *relative* fidelity of different representations can be made without a hard functional–anatomical ground truth that real patient outcome data could never provide: this is because noise in the underlying dependence would unquantifiably modulate any observed effect. The magnitude of the predictive improvement is bound to vary outside idealised conditions, but key here is consistency across a wide array of plausible ground truth models encompassing the entirety of the brain. Real-world prediction needs to be quantified subsequently, only after the limits under ideal condition are established first.

Our approach employs t-SNE, a well-established non-linear dimensionality technique widely regarded as being capable of achieving maximal separation between the clusters of complex distributions (Abdelmoula et al., 2016; Amir et al., 2013; Arazi et al., 2019; Shekhar et al., 2014). But our key conclusion is that non-linear representation learning *in general* is capable of achieving far better predictive power than simple metrics such as lesion volume allow, without introducing complexity in the predictive model itself. The rapidly evolving field of representation learning will bring new methods, and with larger collections of data, established methods such as deep generative models will become tractable. Methods with comparable expressive power but less stochasticity than t-SNE would be desirable, combined with predictive systems downstream within a semi-supervised framework. It is clear that simple, linear methods such as PCA are incapable of accessing the complex structure of lesions (Supplementary Figure 8).

Though a lesion may be assigned to a cluster visually or automatically via distance metrics, by reference to the native-space anatomical appearances of the archetypal centroids, the most natural implementation of our new classification is by automated processing the image, now feasible even with clinical data of variable quality. The fundamental structure of the representation having been established, automated assignment would be performed by a discriminative algorithm, bypassing *t*-SNE whose stochastic nature suits it to the general identification of high-dimensional patterns rather than the categorisation of individual unseen cases (Van Der Maaten, 2009). Comparable disentanglement is in any event achievable with invertible algorithms such as UMAP (Supplementary Figure 8). Rendering the structure intuitively intelligible potentially strengthens clinical trust in algorithms whose operation otherwise appears opaque, even if it may not be in reality.

Though clearly superior to a basic description a lesion, the succinct representation cannot match the predictive performance achievable with a 50-dimensional embedding. Where the available data scale permits it without overfitting, high-dimensional modelling, at maximum relying on voxel-wise information, places a higher ceiling on maximal achievable performance, and remains preferable (Karnath, Sperber, & Rorden, 2018; Mah et al., 2014; Pustina, Avants, Faseyitan, Medaglia, & Coslett, 2018; Toba et al., 2017; Xu et al., 2017). Equally, where the constraint on compactness may be relaxed beyond the naturally intuitive, representations of intermediate dimensionality can be used. This is especially true of lesion-deficit models intended for functional anatomical inference, whose aim is to render the underlying functional anatomy perspicuous rather than to identify conjunctions of lesion and functional anatomical patterns that best predict individual outcomes (Bonkhoff, Lim, et al., 2021; Bonkhoff, Schirmer, et al., 2021; Zhao, Halai, & Lambon Ralph, 2020). Indeed, employing higher dimensionalities is essential if the underlying functional architecture is to be explicitly disentangled from the lesion patterns used to reveal it (Xu, Jha, & Nachev, 2018). Rather, our aim here is to facilitate—through the use of intuitively intelligible representations–the transition to more complex outcome modelling in stroke, where established practice remains aggressively reductive.

Our representation is limited to acute ischaemic stroke: given sufficient data, analogous representations may be derived for chronic lesions, of vascular and other aetiologies whose manifestations are spatially structured. Though here confined to anatomical features, multimodal information can be brought into play if interaction with the anatomy falls within the realm of possibility. Indeed, the integration of other anatomical factors—such as white matter connectivity (Foulon et al., 2018)—and functional indices—such as networks of task-related co-activation (Eickhoff et al., 2018)—as well as their interaction (Thiebaut de Schotten, Foulon, & Nachev, 2020), may well enable succinct representations with higher individually predictive power.

## Credit author statement

**Anna K. Bonkhoff:** Conceptualization, Methodology, Software, Validation, Formal analysis, Writing – Original, Writing - Review & Editing, Visualization; **Tianbo Xu:** Software, Validation, Resources, Writing - Review & Editing; **Amy Nelson:** Software, Validation, Formal analysis, Writing - Review & Editing; **Robert Gray:** Conceptualization, Methodology, Formal analysis, Writing - Review & Editing; **Ashwani Jha:** Conceptualization, Formal analysis, Writing - Review & Editing; **Jorge Cardoso:** Writing - Review & Editing, Funding acquisition; **Sebastien Ourselin:** Funding acquisition, **Geraint Rees:** Writing - Review & Editing, Funding acquisition **Hans Rolf Jäger:** Conceptualization, Methodology, Writing – Original, Writing - Review & Editing; **Parashkev Nachev:** Conceptualization, Methodology, Formal analysis, Resources, Writing – Original, Writing - Review & Editing, Visualization, Supervision, Funding acquisition.

## Funding

This work has been funded by the 10.13039/100010269Wellcome Trust and the UCLH NIHR Biomedical Research Centre.

## Declaration of competing interest

None.

## Footnotes

2 The propensity for dysfunction of a neural substrate to manifest symptomatically.
