# Supplementary material for: Reclassifying stroke lesion anatomy
Source: Cortex. 2021 Dec;145:1–12. doi: 10.1016/j.cortex.2021.09.007 (PMC8650827; doi:10.1016/j.cortex.2021.09.007)
Supplement: Multimedia component 1 [file mmc1.pdf]

# Supplementary Material

## Imaging: detailed methods

*Data acquisition*

All acquisitions were performed on scanners manufactured by General Electric (Genesis Signa), Philips (Achieva and Ingenia), or Siemens (Avanto, Skyra and Verio) with field strength of either 1.5 or 3 Tesla. This diversity reflects changes in routine clinical practice over the period of data collection rather than differences in individual indications. All scans were obtained as part of the clinical routine, employing clinical protocols.

We extracted from each imaging study the echoplanar diffusion-weighted (DWI) for lesion segmentation and inter-subject registration. DWI is widely used to detect and locate acute ischemic lesions(Fiebach *et al.*, 2002). In its clinical application, it consists of an image with a b value of 0 s/mm² that is relatively insensitive to acute ischaemia but shows reasonable tissue contrast, and an image with a b value of 1000 s/mm² that is sharply sensitive to ischaemia but has poor normal tissue contrast. This complementarity can be exploited to achieve both good lesion segmentation—which depends on the contrast between lesioned and normal tissue—and good brain registration—which depends on the contrast between normal tissue types.

*Image pre-processing*

All processing was performed within MATLAB (Mathworks, USA), following a processing pipeline built principally on SPM12 (http://www.fil.ion.ucl.ac.uk/spm/software/spm12/). After conversion from DICOM to NIfTI file format, each image was signal clamped to between 0.1% and 99.9% of the empirical signal distribution estimated with a robust kernel density method (Botev *et al.*, 2010). The images were then denoised with an oracle-based 3D discrete cosine transform filter (Manjón *et al.*, 2012). Each image was then rigidly co-registration to the standard SPM12 tissue probability map in MNI space using normalised mutual information with adjustment from a Procrustes analysis weighted by the white and grey matter compartments (Jha *et al.*, 2016). This rigid procedure was naturally robust to the presence of a lesion in all sequences.

So as to optimise the alignment between the b0 and b1000 images, the former was rigidly co-registered to the latter using SPM's standard co-registration routine with default settings. SPM12's normalisation/segmentation routine(Ashburner and Friston, 2005) was then applied to the b0 image so as to derive a deformation field describing the optimal non-linear transformation into standard Montreal Neurological Institute (MNI) stereotactic space. The parameters of the routine were set at default. This deformation field was then used to transform the b1000 image into MNI space, resliced to 2mm$^3$ isotropic with 4$^{th}$ degree b-spline interpolation (Mah *et al.*, 2012, 2014). Each image was manually checked against the SPM template to confirm satisfactory registration.

*Lesion Segmentation*

With all b1000 images in anatomical register we proceeded to segment each lesion by the voxel-wise application of our previously validated method based on the anomaly metric, *zeta* (Mah *et al.*, 2012). We began by normalising the signal distribution of each individual image by subtracting from it the peak of the white matter signal distribution for that specific image. This was derived from a kernel density estimate (Botev *et al.*, 2010) applied to voxels falling within a custom-made mask constructed by thresholding SPM12's white matter tissue map at a probability greater than 0.9 and excluding areas in the frontal and temporal poles commonly prone to artefact on DWI sequences. Where the radiological report identified a unilateral lesion—the vast majority—the mask was applied only to the unaffected hemisphere; where both hemispheres were affected the mask was applied to both hemispheres. Since ischaemic lesions tend to be small where bilateral, and the kernel density estimate is relatively robust to outliers, the signal normalisation was equally effective in both cases. This normalisation step enabled us to adjust for global differences in the signal distribution across all scans.

For each image, independently for each voxel within the brain, we then calculated the *zeta* anomaly metric against a set of 492 reference images—processed in exactly the same way—derived from patients suspected of stroke in whom no acute lesion was reported by the reviewing radiologist. The zeta value of a voxel in the test image is given by the mean distance to the *k* nearest neighbours drawn from the anatomically homologous voxels in the unlesioned reference set, normalised by the mean distance between the *k* neighbours themselves (Mah *et al.*, 2012). The distance measure here is simply the difference in the voxel signal. This approach makes the metric robust to badly behaved signal distributions, including non-monotonic ones differing substantially in density across voxels. The metric has only one parameter, *k,* here set at 30 based on previous work(Mah *et al.*, 2012, 2014).

The resultant voxel-wise *zeta* maps are real-numbered and dimensionless. In order to threshold them into the binary maps in customary use—denoting each point in the brain as being simply affected or unaffected—we applied a cluster-level thresholding procedure modelling the distribution of *zeta* values within each image under a generalized extreme value (GEV) distribution. For each cluster in each segmented lesion, the estimated GEV mean and variance were used to derive an adaptive threshold, as described in detail and validated elsewhere (Mah *et al.*, 2012).

Supplementary Table 1. Predictive performance with Yeo–Schaefer parcellation. Confidence intervals are given in brackets after each value. Values are derived from cross-validation.

| Network | N affected | Baseline accuracy | Baseline AUROC | Cluster accuracy | Cluster AUROC | 2D accuracy | 2D AUROC | 50D accuracy | 50D AUROC |
|---|---|---|---|---|---|---|---|---|---|
| Motor A | 128 | 0.72 (0.03) | 0.83 (0.01) | 0.82 (0.03) | 0.92 (0.01) | 0.83 (0.02) | 0.92 (0.01) | 0.91 (0.02) | 0.97 (0.006) |
| Motor B | 265 | 0.78 (0.01) | 0.89 (0.01) | 0.84 (0.01) | 0.93 (0.01) | 0.86 (0.01) | 0.94 (0.01) | 0.95 (0.002) | 0.99 (0.001) |
| Peripheral Vision | 88 | 0.70 (0.04) | 0.77 (0.01) | 0.84 (0.03) | 0.92 (0.01) | 0.89 (0.01) | 0.94 (0.01) | 0.97 (0.01) | 0.99 (0.003) |
| Central Vision | 97 | 0.77 (0.05) | 0.83 (0.03) | 0.85 (0.02) | 0.94 (0.01) | 0.87 (0.03) | 0.94 (0.01) | 0.97 (0.01) | 0.99 (0.003) |
| Ventral Attention A | 210 | 0.85 (0.01) | 0.93 (0.01) | 0.87 (0.02) | 0.94 (0.01) | 0.87 (0.02) | 0.95 (0.004) | 0.94 (0.01) | 0.99 (0.002) |
| Ventral Attention B | 66 | 0.85 (0.04) | 0.91 (0.02) | 0.85 (0.03) | 0.92 (0.02) | 0.87 (0.03) | 0.93 (0.01) | 0.93 (0.02) | 0.97 (0.009) |
| Dorsal Attention A | 69 | 0.79 (0.03) | 0.86 (0.02) | 0.86 (0.03) | 0.92 (0.01) | 0.87 (0.03) | 0.95 (0.01) | 0.94 (0.01) | 0.98 (0.007) |
| Dorsal Attention B | 145 | 0.80 (0.02) | 0.89 (0.01) | 0.89 (0.01) | 0.95 (0.01) | 0.87 (0.01) | 0.95 (0.004) | 0.94 (0.01) | 0.99 (0.002) |
| Control A | 183 | 0.84 (0.03) | 0.92 (0.01) | 0.89 (0.02) | 0.95 (0.004) | 0.90 (0.02) | 0.97 (0.01) | 0.96 (0.01) | 0.99 (0.001) |
| Control B | 73 | 0.82 (0.03) | 0.92 (0.01) | 0.88 (0.03) | 0.95 (0.01) | 0.88 (0.04) | 0.95 (0.01) | 0.95 (0.01) | 0.98 (0.007) |
| Control C | 25 | 0.65 (0.1) | 0.73 (0.04) | 0.75 (0.1) | 0.85 (0.03) | 0.82 (0.4) | 0.89 (0.02) | 0.89 (0.03) | 0.96 (0.019) |
| Default A | 103 | 0.84 (0.03) | 0.92 (0.01) | 0.88 (0.02) | 0.96 (0.01) | 0.87 (0.02) | 0.95 (0.01) | 0.94 (0.01) | 0.98 (0.004) |
| Default B | 72 | 0.89 (0.03) | 0.95 (0.01) | 0.90 (0.02) | 0.97 (0.01) | 0.92 (0.02) | 0.97 (0.01) | 0.95 (0.01) | 0.99 (0.004) |
| Default C | 49 | 0.77 (0.04) | 0.82 (0.03) | 0.81 (0.03) | 0.89 (0.02) | 0.83 (0.03) | 0.94 (0.01) | 0.92 (0.01) | 0.99 (0.006) |
| Temporal Parietal | 108 | 0.82 (0.03) | 0.91 (0.01) | 0.83 (0.03) | 0.92 (0.01) | 0.82 (0.02) | 0.91 (0.01) | 0.94 (0.01) | 0.99 (0.005) |
| Limbic | 0 | - | - | - | - | - | - | | |

Supplementary Table 2. Predictive performance with Rorden–Archer parcellation. Confidence intervals are given in brackets after each value. Values are derived from cross-validation.

| Area | N affected | Baseline accuracy | Baseline AUROC | Cluster accuracy | Cluster AUROC | 2D accuracy | 2D AUROC | 50D accuracy | 50D AUROC |
|---|---|---|---|---|---|---|---|---|---|
| Corticospinal Tract | 146 | 0.86 (0.01) | 0.94 (0.01) | 0.90 (0.01) | 0.96 (0.01) | 0.90 (0.01) | 0.97 (0.01) | 0.96 (0.01) | 0.99 (0.002) |
| Sensorimotor Areas | 141 | 0.82 (0.03) | 0.91 (0.01) | 0.88 (0.2) | 0.94 (0.004) | 0.88 (0.2) | 0.95 (0.01) | 0.94 (0.01) | 0.99 (0.003) |
| Vision | 75 | 0.79 (0.04) | 0.84 (0.01) | 0.86 (0.03) | 0.94 (0.01) | 0.89 (0.02) | 0.96 (0.01) | 0.95 (0.01) | 0.99 (0.001) |
| Speech | 221 | 0.86 (0.01) | 0.93 (0.004) | 0.89 (0.02) | 0.96 (0.01) | 0.92 (0.01) | 0.97 (0.01) | 0.95 (0.01) | 0.99 (0.003) |
| Visuospatial Neglect | 175 | 0.84 (0.01) | 0.93 (0.01) | 0.88 (0.02) | 0.95 (0.01) | 0.91 (0.02) | 0.96 (0.01) | 0.96 (0.01) | 0.99 (0.002) |
| Picture Naming | 64 | 0.79 (0.05) | 0.81 (0.02) | 0.84 (0.03) | 0.95 (0.02) | 0.89 (0.03) | 0.96 (0.01) | 0.96 (0.01) | 0.99 (0.004) |

Supplementary Table 3. Detailed parameters of the ANOVA from Rorden-Archer parcellation-based analyses.

| Network | Sum of squares | Degrees of freedom | F-Statistic | Significance | $n^2$ | $\omega^2$ |
|---|---|---|---|---|---|---|
| Main effect: Modelling approach | 0.499 | 3 | 352.204 | 6.991e-83 | 0.727 | 0.724 |
| Main effect: Lesion syndrome | 0.040 | 5 | 16.984 | 3.741e-14 | 0.058 | 0.055 |
| Interaction effect: Modelling*Syndrome | 0.046 | 15 | 6.467 | 2.85e-11 | 0.066 | 0.056 |
| Residuals | 0.102 | 162 | | | | |

Supplementary Table 4. Detailed parameters of the ANOVA from Yeo-Schaefer parcellation-based analyses.

| Network | Sum of squares | Degrees of freedom | F-Statistic | Significance | $n^2$ | $\omega^2$ |
|---|---|---|---|---|---|---|
| Main effect: Modelling approach | 1.594 | 3 | 558.006 | 5.697e-165 | 0.520 | 0.519 |
| Main effect: Lesion syndrome | 0.633 | 14 | 47.462 | 1.423e-84 | 0.206 | 0.202 |
| Interaction effect: Modelling*Syndrome | 0.323 | 42 | 8.086 | 1.395e-35 | 0.106 | 0.092 |
| Residuals | 0.514 | 540 | | | | |

Supplementary Figures

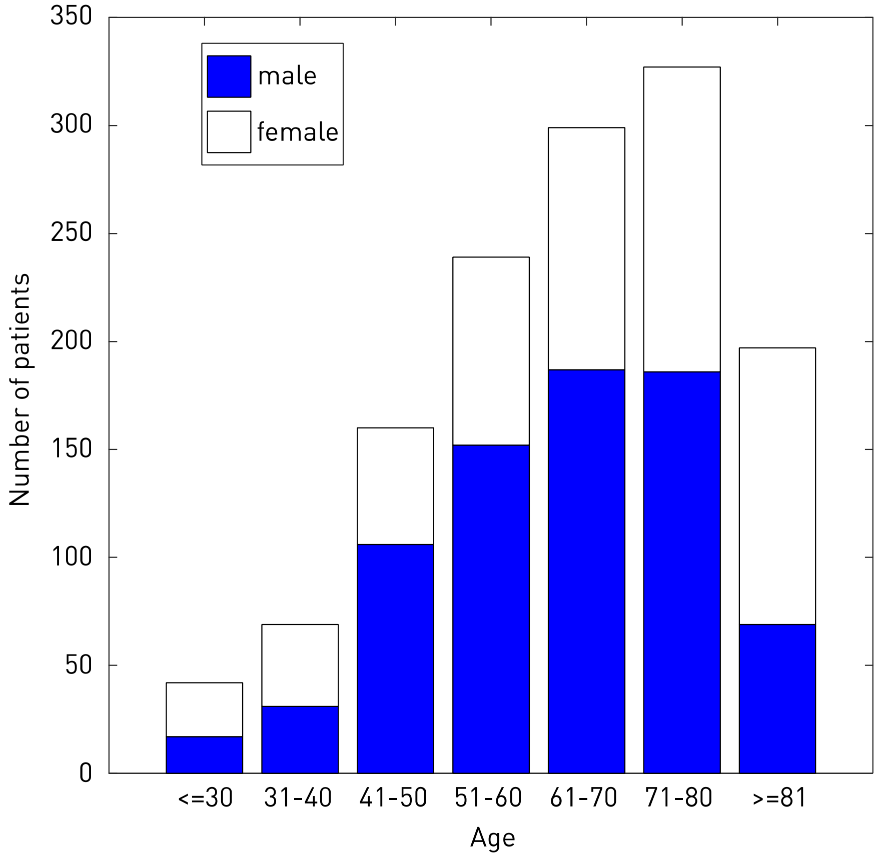


Supplementary Figure 1. Age and sex distribution as available for 88 % of the stroke subjects included.

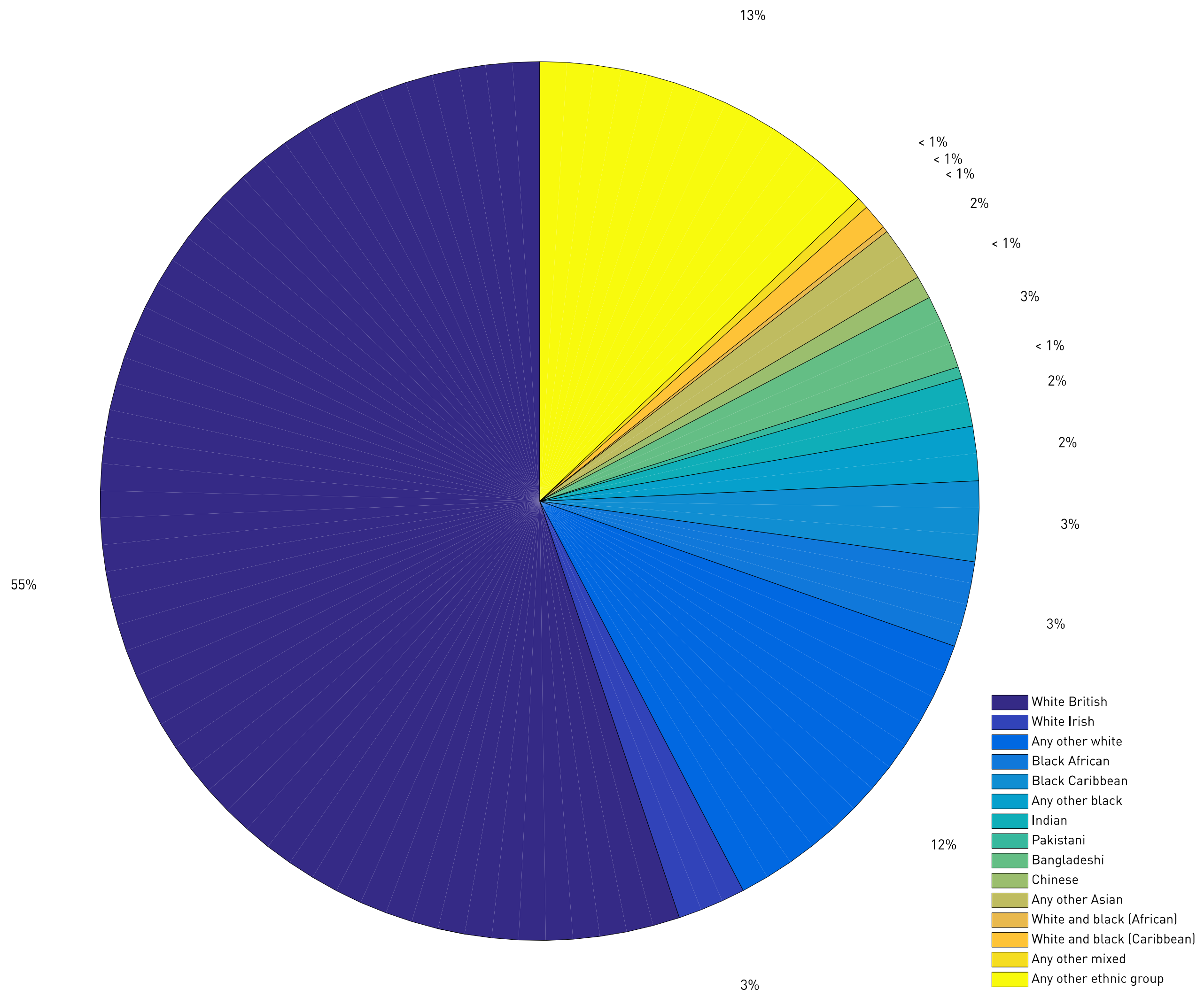


Supplementary Figure 2. The distribution of self-reported patient ethnicity for the 62% of patients in whom it was recorded.

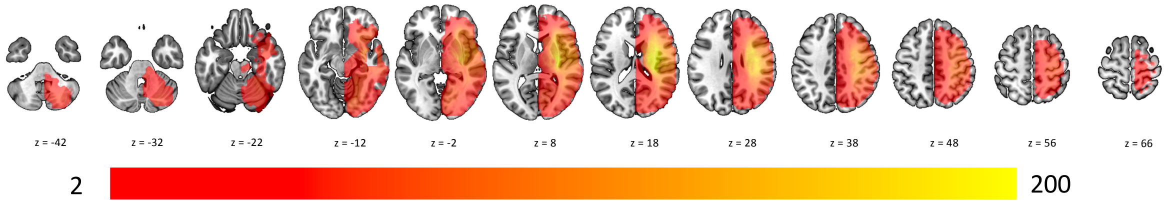


Supplementary Figure 3. Overlay map of all (n=1333) lesions. The colour map indicates the number of subjects with a lesion at each specific voxel.

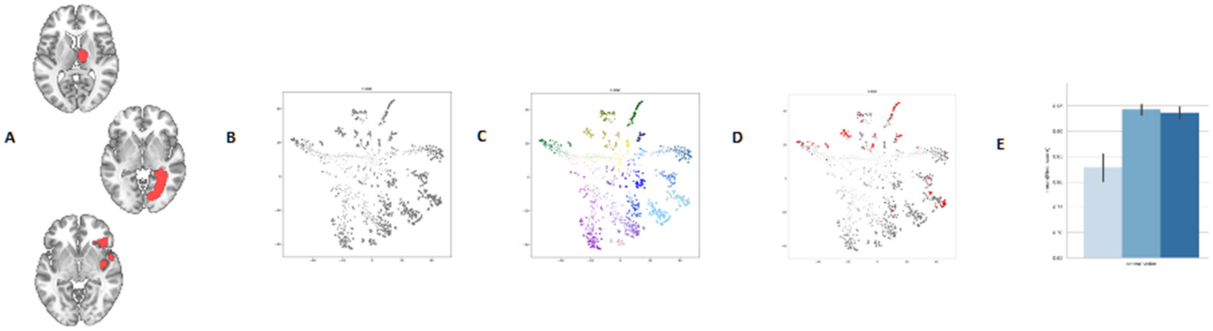


Supplementary Figure 4. Outline of the entire workflow. A. 1333 binary ischaemic stroke lesion maps are defined in a 902 629 dimensional voxel space at resolution 2x2x2 mm$^3$; illustrated here are three examples. B. Two-dimensional representation of the 1333 lesion maps obtained with *t*-stochastic neighbour embedding following dimensionality reduction step to 50 dimensions with non-negative matrix factorization. C. Agglomeration of the resultant embedding into 21 final clusters. D. Identification of embedded lesions overlapping with a target anatomical network: here visual regions as defined by Brodmann areas 17, 18 and 19. E. Cross-validated accuracy of models attempting to predict whether or not a hypothetical syndrome associated with damage to the target region is present or absent in a given patient. Gradient boosting machine classifiers are trained on conventional input features (light blue: subject age & lesion volume only), or the new lesion features we investigate here (blue: subject age & lesion volume & t-SNE cluster, dark blue: subject age & lesion volume & t-SNE-coordinates).

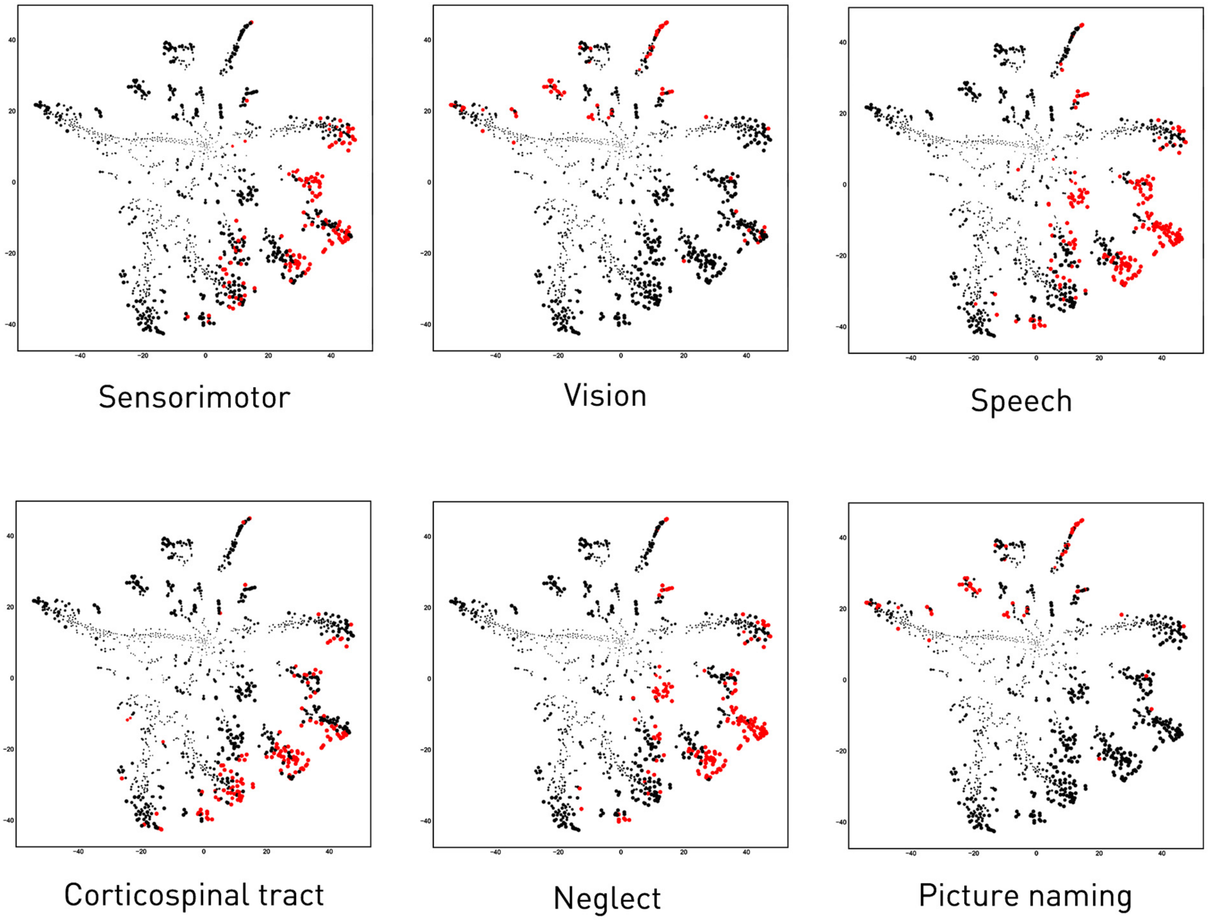


Supplementary Figure 5. t-SNE visualization of hypothetical lesion syndromes based on Rorden-Archer parcellation. Those hypothetically affected by the lesion are marked in red.

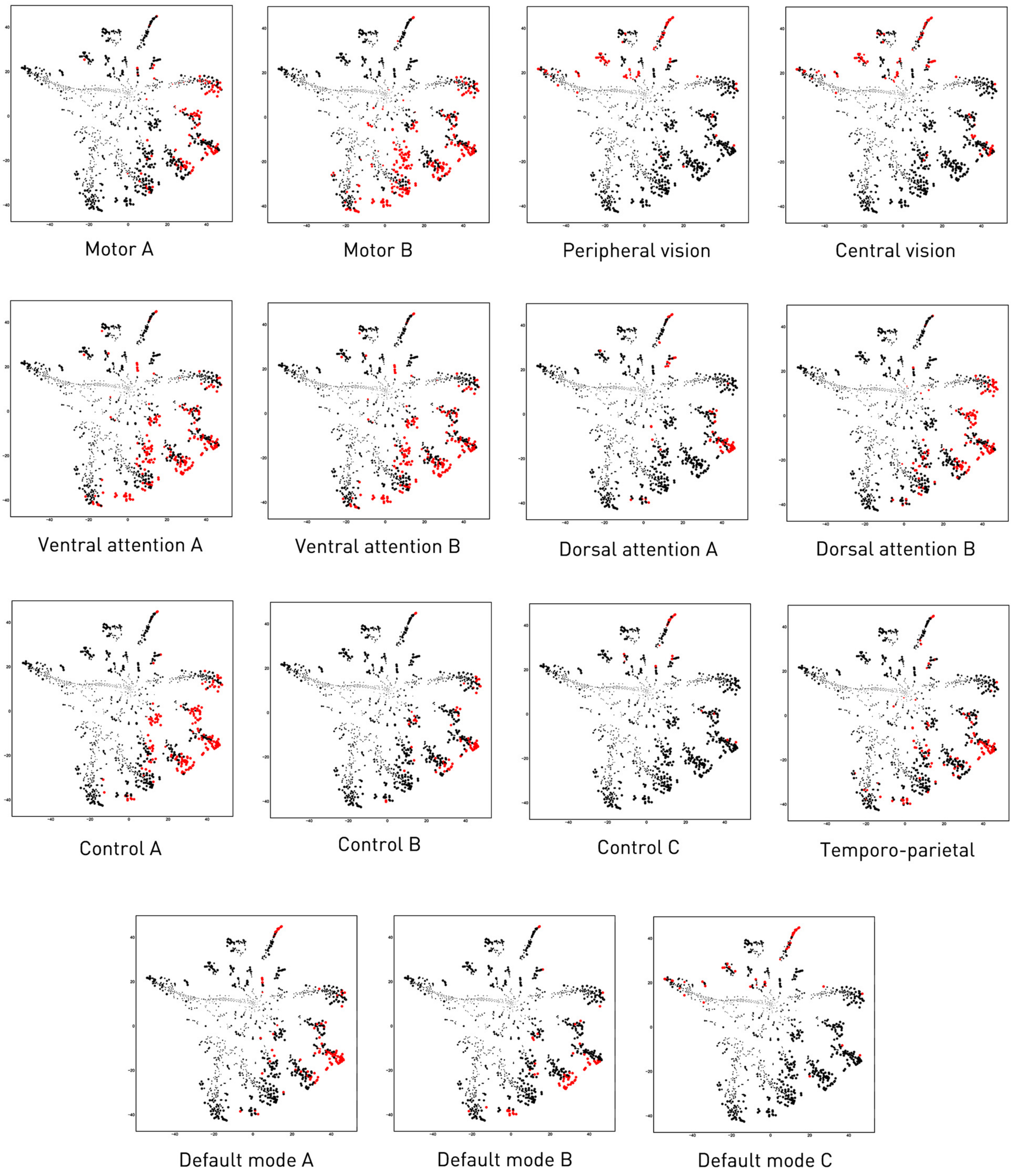


Supplementary Figure 6. t-SNE visualization of hypothetical lesion syndromes based on Yeo–Schaefer parcellation. Those hypothetically affected by the lesion are marked in red.

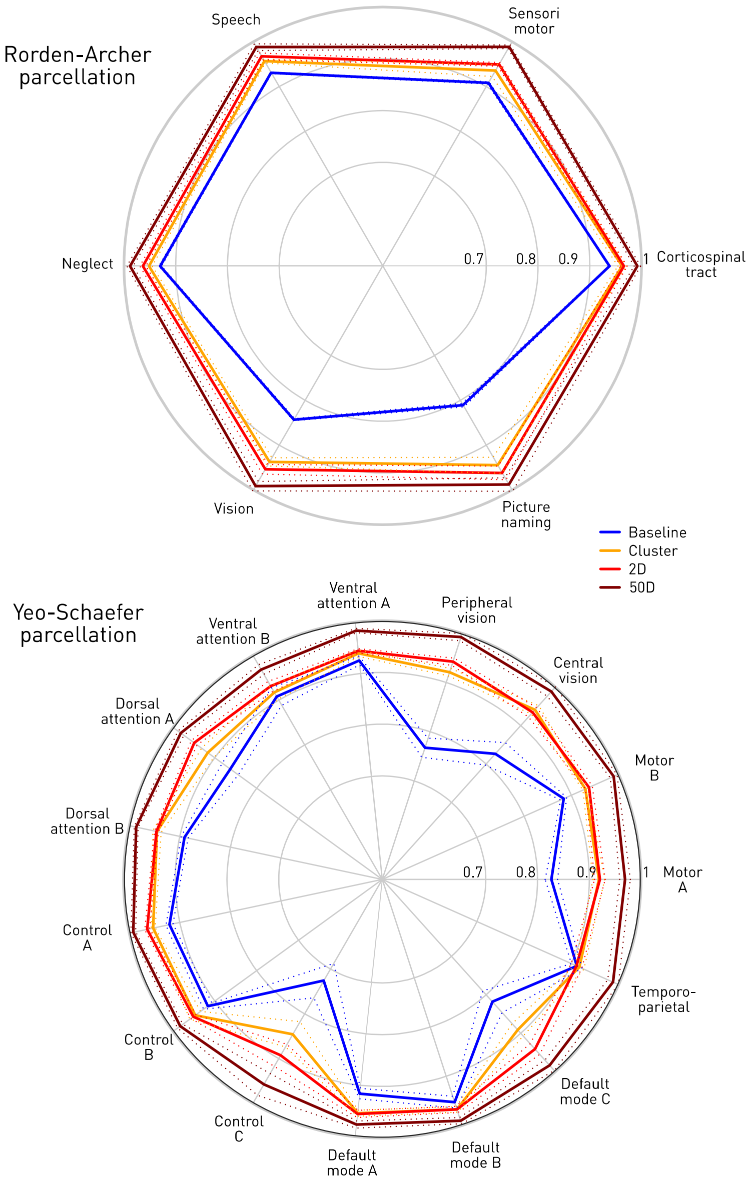


Supplementary Figure 7. Quantification of simulated behavioural outcome predictive performance. For each of four incrementally enriched representations—baseline age and lesion volume (blue), cluster membership (orange), two-dimensional representation coordinates (red), and fifty-dimensional NMF representations coordinates (claret)—achieved AUROC is depicted as a spider-plot across individual areas within the Rorden-Archer parcellation (top), and the Yeo-Schaefer parcellation (bottom). Dotted lines identify 95% confidence intervals from the cross-validation procedure. The origin of the spider indicates prediction at chance level (50%); outer circles indicate 70%, 80% and 90% accuracy. Note that predictive accuracy generally increases with dimensionality but that the 21 cluster representation performs substantially better than age and lesion volume alone.

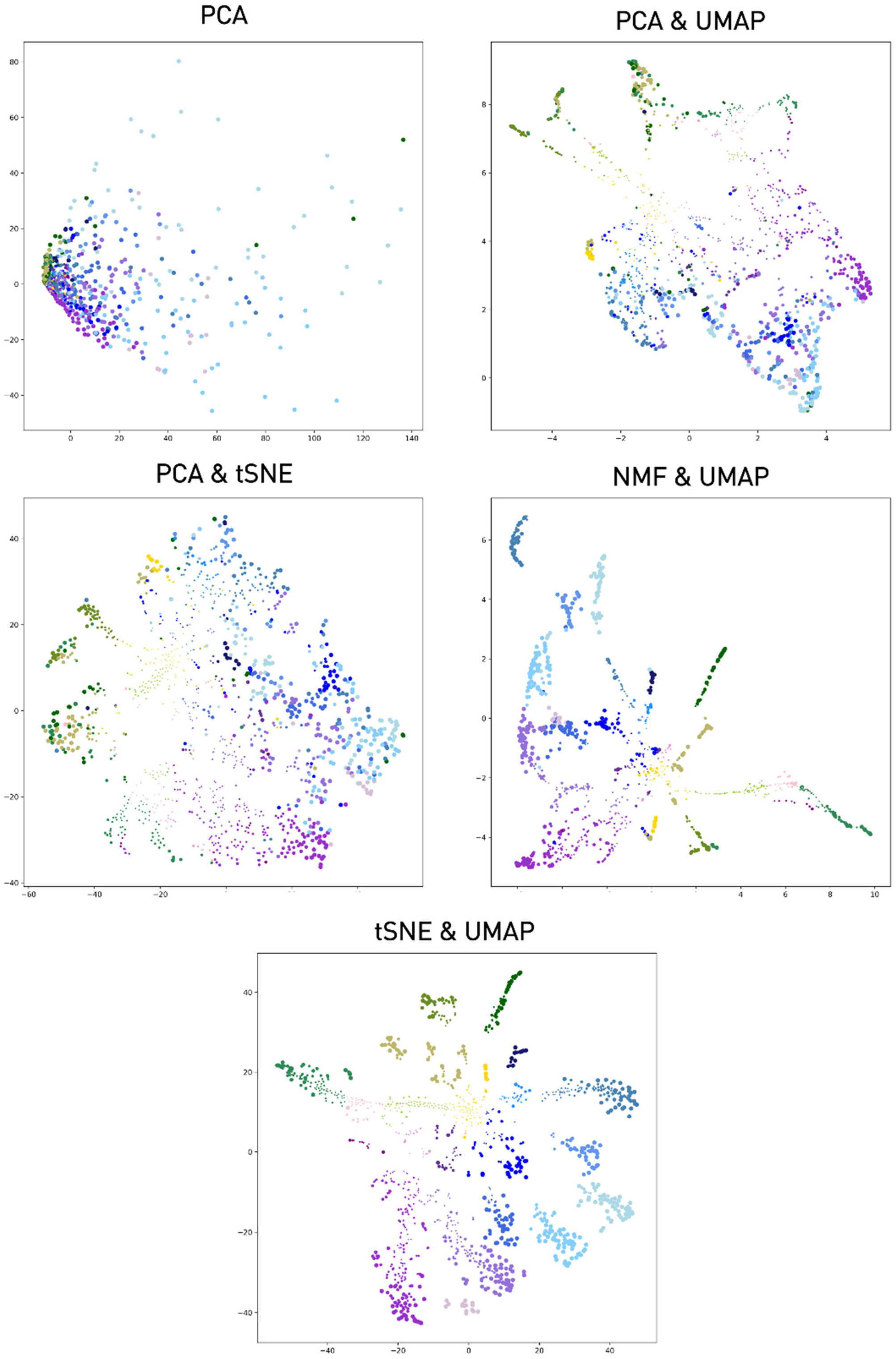


Supplementary Figure 8. Further low dimensional embeddings. Marker size varies with lesion size. Colour coding corresponds to the 21 cluster labels as inferred from the low-dimensional embedding via non-negative matrix factorization (NMF) and t-distributed stochastic neighbour embedding (t-SNE) utilized for the main analysis (replicated in the last panel). Note the similarity between UMAP and t-SNE.

## Supplementary References


Ashburner J, Friston KJ. Unified segmentation. Neuroimage 2005; 26: 839–851.

Botev ZI, Grotowski JF, Kroese DP. Kernel density estimation via diffusion. The Annals of Statistics 2010; 38: 2916–2957.

Corbetta M, Akbudak E, Conturo TE, Snyder AZ, Ollinger JM, Drury HA, et al. A Common Network of Functional Areas for Attention and Eye Movements. Neuron 1998; 21: 761–773.

Fiebach JB, Schellinger PD, Jansen O, Meyer M, Wilde P, Bender J, et al. CT and Diffusion-Weighted MR Imaging in Randomized Order. Stroke 2002; 33: 2206–2210.

Group TN t-PSS. Generalized Efficacy of t-PA for Acute Stroke. Stroke 1997; 28: 2119–2125.

Jha A, Diehl B, Scott C, McEvoy AW, Nachev P. Reversed Procrastination by Focal Disruption of Medial Frontal Cortex. Current Biology 2016; 26: 2893–2898.

Mah Y-H, Husain M, Rees G, Nachev P. Human brain lesion-deficit inference remapped. Brain 2014: awu164.

Mah Y-H, Jager R, Kennard C, Husain M, Nachev P. A new method for automated high-dimensional lesion segmentation evaluated in vascular injury and applied to the human occipital lobe. Cortex 2012

Manjón JV, Coupé P, Buades A, Louis Collins D, Robles M. New methods for MRI denoising based on sparseness and self-similarity. Medical Image Analysis 2012; 16: 18–27.

Muir KW, Weir CJ, Murray GD, Povey C, Lees KR. Comparison of Neurological Scales and Scoring Systems for Acute Stroke Prognosis. Stroke 1996; 27: 1817–1820.

Sindhwani V, Keerthi SS. Large Scale Semi-supervised Linear SVMs. In: Proceedings of the 29th Annual International ACM SIGIR Conference on Research and Development in Information Retrieval. New York, NY, USA: ACM; 2006. p. 477–484.
